# Spontaneous Facial Mimicry is Modulated by Joint Attention and Autistic Traits

**DOI:** 10.1002/aur.1573

**Published:** 2015-10-07

**Authors:** Janina Neufeld, Christina Ioannou, Sebastian Korb, Leonhard Schilbach, Bhismadev Chakrabarti

**Affiliations:** ^1^ Centre for Integrative Neuroscience and Neurodynamics, School of Psychology and Clinical Language SciencesUniversity of ReadingReadingUK; ^2^ Department of Women's and Children's HealthCenter of Neurodevelopmental Disorders at Karolinska Institutet (KIND)StockholmSweden; ^3^ Laboratoire des Neurosciences Cognitives (LNC)INSERM U960, Institut d'Etudes Cognitives, Ecole Normale SupérieureParisFrance; ^4^ Neuroscience and Society Lab, SISSATriesteItaly; ^5^ Department of PsychiatryUniversity Hospital CologneGermany; ^6^ Max‐Planck Institute of PsychiatryMunichGermany

**Keywords:** joint attention, spontaneous facial mimicry, empathy, gaze‐based social interaction, autism

## Abstract

Joint attention (JA) and spontaneous facial mimicry (SFM) are fundamental processes in social interactions, and they are closely related to empathic abilities. When tested independently, both of these processes have been usually observed to be atypical in individuals with autism spectrum conditions (ASC). However, it is not known how these processes interact with each other in relation to autistic traits. This study addresses this question by testing the impact of JA on SFM of happy faces using a truly interactive paradigm. Sixty‐two neurotypical participants engaged in gaze‐based social interaction with an anthropomorphic, gaze‐contingent virtual agent. The agent either established JA by initiating eye contact or looked away, before looking at an object and expressing happiness or disgust. Eye tracking was used to make the agent's gaze behavior and facial actions contingent to the participants' gaze. SFM of happy expressions was measured by Electromyography (EMG) recording over the Zygomaticus Major muscle. Results showed that JA augments SFM in individuals with low compared with high autistic traits. These findings are in line with reports of reduced impact of JA on action imitation in individuals with ASC. Moreover, they suggest that investigating atypical interactions between empathic processes, instead of testing these processes individually, might be crucial to understanding the nature of social deficits in autism. ***Autism Res*** *2016, 9: 781–789*. © 2015 The Authors Autism Research published by Wiley Periodicals, Inc. on behalf of International Society for Autism Research

## Introduction

Autism spectrum conditions (ASC) are neurodevelopmental disorders, characterized primarily by pervasive impairments in social interaction and communication [American Psychiatric Association, 2013]. Atypical spontaneous facial mimicry (SFM) of emotion expressions has been observed in children, adolescents, and adults with ASC [Beall, Moody, McIntosh, Hepburn, & Reed, 2008; Edwards, 2014; McIntosh, Reichmann‐Decker, Winkielman, & Wilbarger, 2006; Oberman, Winkielman, & Ramachandran, 2009]. Spontaneous mimicry is believed to underlie the more primitive, emotional aspects of empathy [Bavelas, Black, Lemery, & Mullett, 1990; Hatfield & Cacioppo, 1994; Plutchik, 1990]. It is present early in infancy [Field, Woodson, Greenberg, & Cohen, 1983; Meltzoff & Moore, 1977], difficult to inhibit voluntarily [Korb, Grandjean, & Scherer, 2010], and provides a direct route to the embodiment of another person's emotional facial expression that can potentially serve as a mechanism for social understanding [Niedenthal, 2007]. Greater SFM is positively correlated with trait emotional empathy in adults [Sonnby‐Borgstrom, Jönsson, & Svensson, 2003]. A recent review of the literature on mimicry elucidates its relationship with emotional contagion, widely believed to be a component of emotional empathy [Chartrand & Lakin, 2013]. The extent to which SFM is modulated by top–down processes [Hamilton, 2015] and/or by one's own emotional state [Hess & Fischer, 2013], as well as its neural correlates [Korb et al., 2015], constitutes an area of active research. Direct eye contact between the sender and the receiver of an emotional facial expression has been suggested to be a trigger for SFM [Niedenthal, Mermillod, Maringer, & Hess, 2010], and modulates the mimicry of hand movements [Wang, Newport, & Hamilton, 2010]. Wang and Hamilton have further suggested that gaze constitutes a critical social cue that modulates mimicry in a top–down fashion, and that this top–down modulation may be atypical in autism [Wang & Hamilton, 2012].

The specific gaze‐related social cue of interest to us in this experiment is joint attention (JA). JA involves the social coordination of two individuals' visual attention toward an aspect of the environment, and is a reliable predictor of Theory of Mind (ToM) and language abilities in childhood [Charman et al., 2000; Striano & Reid, 2006]. It has been demonstrated that children make use of eye gaze information to make inferences about the desires of the interaction partner [Lee, Eskritt, Symons, & Muir, 1998] and that mentalizing networks are activated as an effect of interpersonal gaze coordination, pointing to the view that the latter may trigger mentalizing processes [Schilbach et al., 2010]. To distinguish JA from other gaze‐driven social cues, we use the framework suggested by Nathan Emery [Emery, 2000]. According to this framework, JA is a case of gaze following in which gaze leader and follower direct their focus of attention to the same aspect of the environment, while “Shared Attention” is a special case of JA that is initiated by direct eye contact between gaze leader and gaze follower. This mutual gaze between the leader and the follower is arguably essential for attention to be truly joint [Tomasello, 1995; Tomasello & Carpenter, 2007]. JA is apparent from the first year of age [Scaife & Bruner, 1975], whereby mutual gaze serves as an “ostensive signal,” and, thus, plays a vital role in learning about the environment and subsequent social competence [Csibra & Gergely, 2011; Mundy & Newell, 2007]. A large number of studies have suggested that persons with ASC are impaired in processing gaze direction and consequently in engaging in JA situations [for a review see: Nation & Penny, 2008]. This deficit has been suggested to play a significant part in the developmental etiology of autism [Mundy & Newell, 2007].

Despite a large number of studies on SFM or JA in autism [Bayliss & Tipper, 2005; Beall et al., 2008; McIntosh et al., 2006], the interrelationship of these two processes has not been directly studied. Doing so might be particularly relevant both for a more in‐depth understanding of the behavioral mechanisms of real‐life social interaction, and for an analysis of disorders of social cognition from an interactor's rather than from an observer's point of view [Schilbach, 2014; Schilbach et al. 2013]. In a recent study investigating the effects of gaze direction on the mimicry of hand actions, Vivanti and Dissanayake [2014] found that while Typically Developing (TD) individuals show greater mimicry of hand actions preceded by direct compared with averted gaze [see also: Wang et al., 2010], this difference was not seen in a group of ASC individuals, who instead showed a trend for greater attention to faces with averted compared to direct gaze. Similarly, children with ASC behaved no differently from typically developing controls when asked to imitate an agent with averted gaze, but performed significantly worse than controls in the direct gaze condition [Vivanti et al., 2011]. In contrast, Senju et al. [2009] investigated contagious yawning in ASC and TD children, and found that both groups performed similarly when they were explicitly instructed to look at the eyes of the yawning person.

To directly test the link between the two phenomena (JA and SFM) in relation to autistic traits, this study measured SFM (using facial EMG) in response to JA, using a gaze‐contingent interactive agent. It tests, first, the interrelationship of SFM and JA and second, if this interrelationship is modulated by autistic traits. Notably, in contrast to the gaze direction studies discussed above, where the participant is a passive observer, in this study the participant's gaze behavior (recorded using an eye‐tracker) determines the behavior of the virtual character in real time [Schilbach et al., 2013]. By more closely resembling a real life social interaction, this scenario has high ecological validity, and has previously been successfully used to study JA [Schilbach et al., 2010; Wilms et al., 2010]. We hypothesize that within this interactive paradigm, JA initiated by mutual gaze will facilitate SFM of happy faces. Further, we predict that the extent of this facilitation will be greater for individuals with low compared with those with high autistic traits.

## Methods

### Participants

Sixty‐two young adults (29 females) with no self‐reported psychiatric/neurological condition, drawn from in and around the campus of the University of Reading took part in the experiment. Mean age of the sample was 22 years (range: 18–38 years). All participants gave informed consent prior to participation. The study was approved by the University of Reading Research Ethics Committee.

### Stimuli and Trait Measures

Participants filled in the Autism Spectrum Quotient [AQ; Baron‐Cohen, Wheelwright, Skinner, Martin, & Clubley, 2001] online before taking part in the experiment. The AQ is a 50‐item questionnaire measuring autistic traits. A similar etiology of autistic traits has been found across the diagnostic divide, suggesting the utility of general population‐based samples for studying autism‐related phenotypes [Robinson et al., 2011].

**Figure 1:**
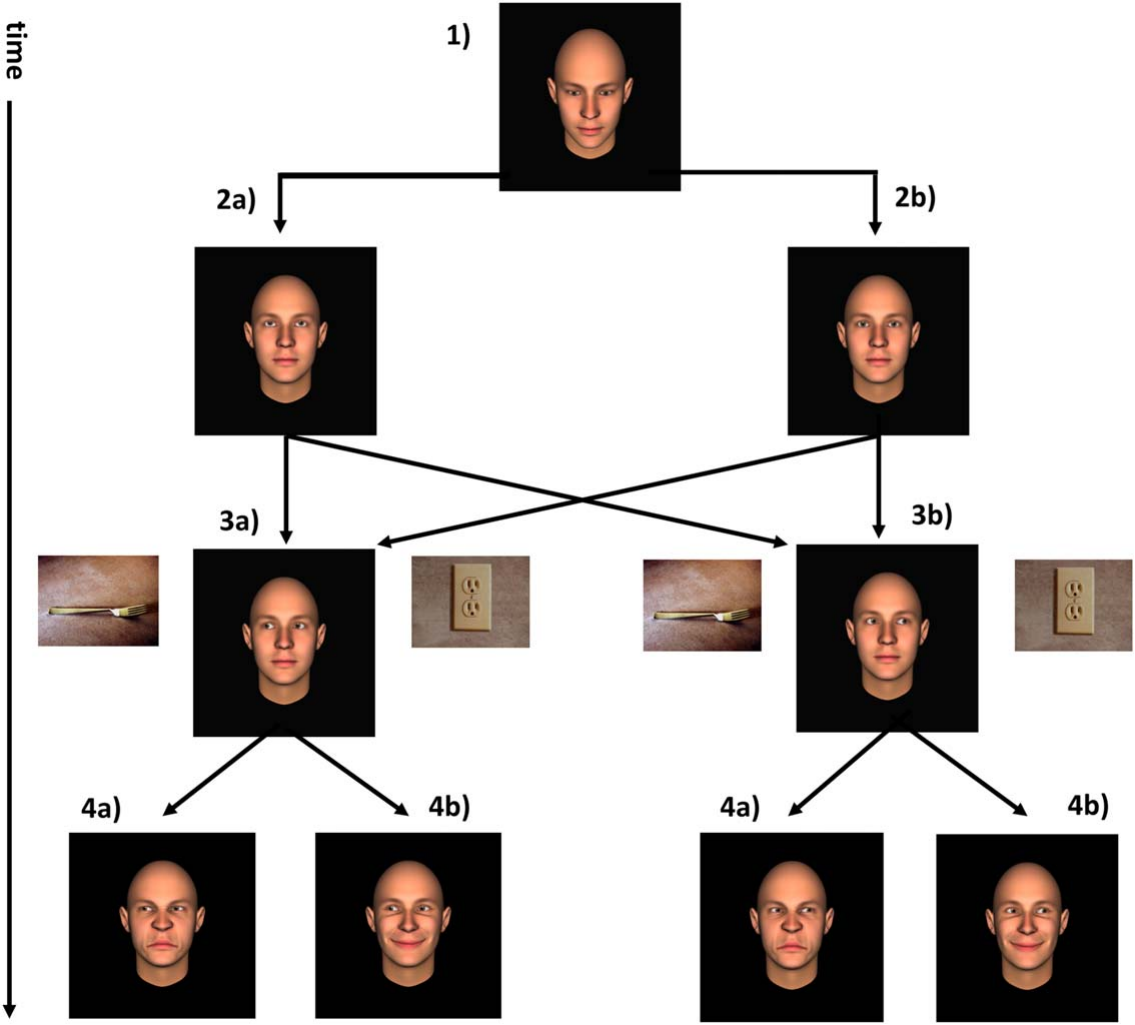
Schematic representation of one trial of the task. (1) The virtual agent looks down at the beginning of the trial. The participant focuses on the eye region of the virtual agent. Then, the virtual agent either averts the gaze (2a) by looking up (No_JA) or engages in eye contact with the participant (JA, 2a). The virtual agent shifts his focus of attention to an object on the left (3a) or right (3a) and the participant follows his gaze. The virtual agent maintains a neutral expression until this point. After the participant's gaze has been detected in the object region, the virtual agent performs a facial expression of either disgust (4a) or happiness (4b) for 1000 ms after participant looked at expression.

An interactive gaze‐contingent paradigm was used to evoke the subjective feeling of JA in the participant [based on Wilms et al., 2010; see Fig. 1]. Stimuli consisted of a male face produced with FACSGen software [Roesch et al., 2011]. The face was centrally presented on a 1152 × 864 pixels screen and scaled to 700 × 700 pixels. The agent could display three different facial expressions: neutral, happy and disgusted, and five gaze directions; either straight gaze or one of four averted gazes (downward, upward, left, or right side horizontally). To serve as targets of the JA focus, pictures of neutral real life objects were chosen from the International affective picture system (IAPS) set [Lang, Bradley, & Cuthbert, 1999] and from publicly available creative commons licensed images on the web. The justification for using neutral objects (e.g. spoons, cups) was to avoid interaction effects with object valence and agent expression as observed by Bayliss, Schuch, and Tipper [2010]. All stimuli were presented using Presentation (version 16.0) software (www.neurobs.com) and eye tracking data was recorded using a Tobii T60 eye tracker (www.tobii.com).

### Procedure

Participants were seated at 55 cm from the screen, which included the eye‐tracker. They were familiarized with the interactive task (Fig. 1), EMG electrodes were attached, and the eye tracker was calibrated. We use the term JA here in the sense of Tomasello and Carpenter [2007] to differentiate between gaze following initiated by mutual gaze (JA) or without preceding eye contact (trials that started with an averted gaze; NoJA). The task had a 2 × 2 design with two conditions of JA and NoJA and emotion expression (the agent either performed a smile in the experimental condition, or a disgusted facial expression in the control condition). The rationale for choosing disgust as control condition was that the expression of disgust toward an object in the environment appeared more likely than the expression of other (more interpersonal) emotions such as sadness or anger. Consequently there were four randomly intermixed trial types; JA_happy, JA_disgust, NoJA_happy, NoJA_disgust. The direction of the agent's gaze (right/left) was fully balanced with respect to the other two conditions. The experiment consisted of 120 trials and lasted for about 1 hr.

In each trial, the agent was presented in central position between two neutral objects, and initially looked down. Participants were instructed to visually fixate on the eye region of the agent. When participants' point of fixation was detected within the eye region of the agent, the agent turned his eyes either to the participant (direct gaze condition for establishment of JA) or upward (averted gaze condition, NoJA). After 1000 ms the agent's eyes turned to either of the two objects on his left and right, thus acting as a directional cue for the participant. Once the participant had followed the gaze to the object that the agent was looking at, after a delay of 500 ms the agent displayed a happy or a disgusted facial expression and displayed it until the participant looked back at the agent and further 1000 ms from then. Following each trial, participants were asked to respond which object they thought the agent liked more. The aim of this preference judgment task was to ensure that participants were attending to the object stimuli as well as to the agent's facial expression.

### Data Acquisition

To provide an index of SFM, EMG activity was recorded throughout the task using ADI Power Lab 8T, with an Octal Bioamp (AD Instruments, Australia), as described in Sims, Van Reekum, Johnstone, and Chakrabarti [2012]. The skin of the participant was cleansed using 70% alcohol prep pads (Professional Disposables, Inc., USA TD‐230) to reduce impedance. Four‐millimeter Ag/AgCl EMG surface sensors (Discount Disposables, USA) on 5 mm collars filled with isotonic electrode gel were attached bipolarly to the participant's left Zygomaticus Major muscle, according to established guidelines [Fridlund & Cacioppo, 1986]. A ground electrode was attached to the participant's forehead.

### Data Handling

EMG data recorded in response to the agent's happy or disgusted facial expression was band pass filtered (50–450 Hz), rectified, and logarithmically transformed to avoid undue influence by extreme values [see Sims et al., 2012]. The pretrial baseline consisted of the mean amplitude of the signal during the 500 ms prior to the onset of the agent's facial expression. Mean signal in response to the expression of the agent was calculated for 1000 ms after the participant looked back at the agent, ensuring that they were attending to the agent's facial expression (Fig. 1). Trials with a baseline exceeding the average baseline amplitude by more than three standard deviations (SDs) were considered as noise and excluded from statistical analysis. EMG data were normalized by dividing each trial's mean signal amplitude by its baseline. Means across all trials per condition (JA‐Happy, NoJA‐Happy, JA‐Disgust, NoJA‐Disgust) were calculated for each participant. Participants whose mean across trials exceeded three SDs of the sample mean for each condition were excluded from the final analyses.

A 2 × 2 repeated measures Analysis of Variance (ANOVA) was conducted to investigate the effect of JA (two levels, JA vs. NoJA) and Emotion (two levels, happy vs. disgusted) on the Zygomaticus Major activity. It is worth noting that only Zygomaticus Major activity in response to the virtual agent's happy as well as disgust expression was recorded. The Zygomaticus Major is essential for making a happy expression but largely irrelevant for making a disgust expression. The latter thus served as a control condition in this experiment to validate that the increased Zygomaticus Major response is an index of greater spontaneous mimicry. Further, to investigate the potential modulation of these effects by participants' autistic traits, a difference score was calculated between the JA‐Happy and NoJA‐Happy conditions. The data were found to be normally distributed (Shapiro–Wilk *P* = 0.801) and, hence, a parametric correlation between the AQ and the JA‐NoJA difference score was computed.

One participant was excluded because his/her overall mean across the four conditions exceeded three SDs from the sample mean for all four conditions. Three more participants were excluded because they had completed less than 80% of the trials due to technical problems. Eight participants were excluded based on the criterion Cook's *d* > 4/*n* (=0.064). In total, twelve participants were excluded from the final analyses (using the cutoff criteria defined above), resulting in a final sample of 50 participants (26 males). AQ data was scored according to guidelines of Baron‐Cohen et al. [2001].

## Results

**Figure 2:**
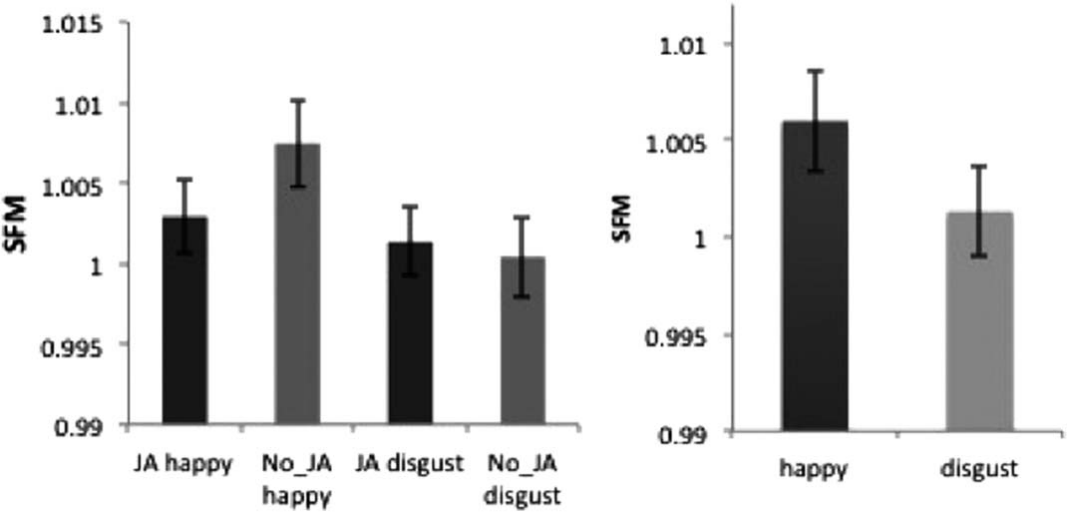
(Left) Zygomaticus Major response in all four experimental conditions. (Right) Average Zygomaticus Major response to happy and disgust conditions (combining JA and no‐JA conditions for each emotion). Error bars indicate 1 standard error of mean (within‐subject).

A 2 × 2 within‐subject ANOVA with two factors (JA and Emotion) and participant gender as a between‐subject control variable showed a trend toward significance for a JA by Emotion interaction (*F*(1, 48) = 3.424, *P* = 0.07). Main effects of JA (*F*(1,48) = 0.279, *P* = 0.6) and Emotion (*F*(1,48) = 0.931, *P* = 0.34), and gender (F(1.48) = 1.931, *P* = 0.171) were not significant (Fig. 2).

**Figure 3:**
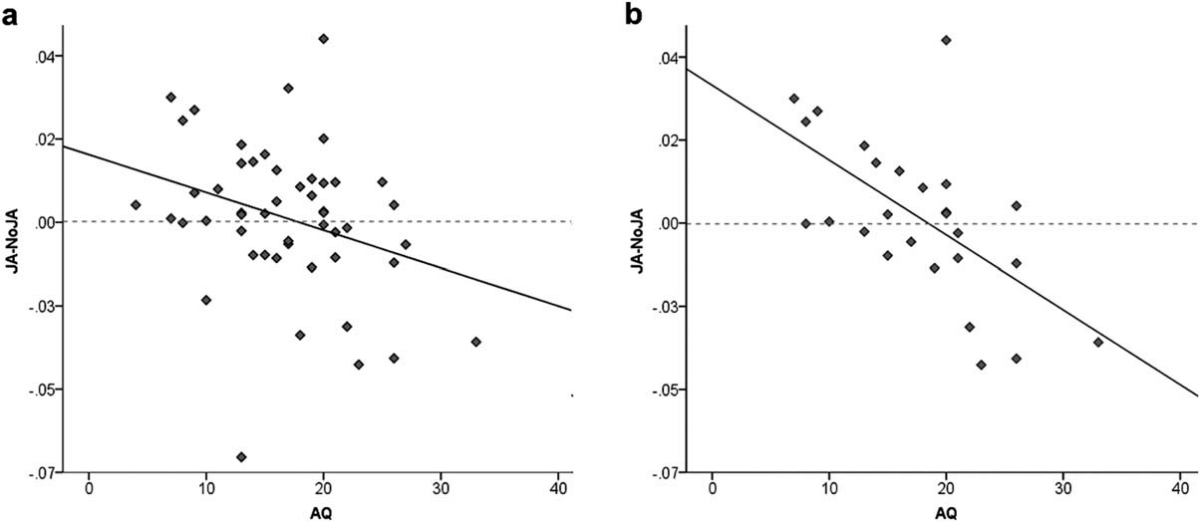
Inverse correlation between AQ and the difference score between JA‐Happy and NoJA‐Happy in Zygomaticus Major activity (JA‐NoJA) in the (a) whole sample, and (b) in male participants only.

To investigate if the JA by Emotion interaction is modulated by autistic traits, a difference score was calculated between the JA‐Happy and NoJA‐Happy conditions. This difference score was significantly negatively correlated with the AQ (*r* = −0.304, *P* = 0.032, Fig. 3a). This effect was driven primarily by males (*r* = −0.611, *P* = 0.001, Fig. 3b) and did not reach significance in females (*r* = 0.115, *P* = 0.592). To test for the existence of a ceiling effect in either AQ or the difference score, we compared the AQ and JA‐NoJA‐Happy scores between the two genders. This analysis revealed no significant difference for either variable (AQ: *t*(48) = −1.115, *P* = 0.270; JA‐NoJA‐Happy: *t*(48) = −0.119, *P* = 0.906).

## Discussion

The effect of JA on SFM of happy faces was tested using a socially interactive paradigm where the participant was involved in gaze‐based JA with an anthropomorphic virtual agent. In particular, we tested whether the relationship of JA and SFM was modulated by autistic traits. The hypothesis that JA increases SFM was not supported, as the JA by Emotion interaction only reached trend level. Crucially however, it was observed that the increase of SFM through JA was modulated by autistic traits, that is, individuals low in autistic traits showed a greater facilitation of SFM by JA.

The interaction between JA and SFM showed a marginal trend for the Zygomaticus Major response to happy faces to be higher in the NoJA condition. This trend is in a direction opposite to what we expected based on previous studies [Brugger, Lariviere, Mumme, & Bushnell, 2007; Wang et al., 2010]. A closer look reveals that this trend is driven by individuals with higher AQ scores. This result is particularly evident by the negative correlation between the JA‐NoJA difference score and AQ. This negative correlation with autistic traits suggests that individuals low in autistic traits mimicked happy faces more in the JA condition, while individuals high in autistic traits mimicked the happy faces more in the condition where JA was not established (NoJA). This is consistent with the finding that children with ASC, unlike controls, fail to increase attention to the model and imitation of her goal‐directed actions when preceded by direct compared to averted gaze [Hamilton, 2015; Vivanti & Dissanayake, 2014]. The latter observation has been interpreted as evidence to support the hypothesis that children with ASC might not automatically prioritize information associated with direct gaze and consequently fail to use this social cue to understand what and when to imitate. This result also corroborates findings from electrophysiological and neuroimaging studies, which have shown atypical neuronal responses to direct gaze in infants with ASC or their baby siblings [Elsabbagh et al., 2009; Grice et al., 2005] as well as in adults with ASC [Georgescu et al. 2013; Kliemann, Dziobek, Hatri, Baudewig, & Heekeren, 2012], suggesting an atypical processing of direct gaze in ASC [Frischen, Bayliss, & Tipper, 2007; Senju & Johnson, 2009].

The negative correlation between the JA‐NoJA difference score and autistic traits lends itself to several explanations, which are not mutually exclusive. First, the suggested effect of JA on altering the reward value of faces [Schilbach et al., 2010] is not seen in individuals high in autistic traits. Second, JA alters the reward value of the face in individuals high in autistic traits, but this increased reward value does not modulate SFM. This explanation is consistent with a previous study, where it was observed that individuals high in autistic traits do not show greater SFM for more rewarding faces [Sims et al., 2012]. A third explanation pertains to the observed tendency in individuals with high AQ to mimic more in the NoJA condition (note that the regression line crosses zero on the *y*‐axis; Fig. 3). This observation is consistent with that reported in children with ASC, who were as accurate in imitating actions as TD children when the model's gaze was averted, but performed worse than the TD group in the direct gaze condition [Vivanti et al., 2011]. It is possible that the direct gaze situation induces more anxiety in individuals high in autistic traits, which can inhibit SFM of happy expressions [Vrana & Gross, 2004]. Future experiments should attempt to explicitly test these possibilities against each other. Importantly, the current observations cannot be due to the fact that individuals high in autistic traits are poorer in distinguishing direct from averted gaze and consequently JA from NoJA. Indeed, the task used in this study required participants to look at the eyes of the agent during the JA/NoJA initiation phase (i.e., the task would not proceed unless a fixation to the eye region was recorded).

Mimicry of facial expressions as well other body movements (such as gestures and postures) is essential for social interaction and has been found to be associated with affiliation and liking [Lakin & Chartrand, 2003; Lakin, Jefferis, Cheng, & Chartrand, 2003; McIntosh, 2006]. Further, SFM can potentially serve as a mechanism for understanding emotions and mental states of others by simulating them [Korb, Niedenthal, Kaiser, & Grandjean, 2014; Künecke, Hildebrandt, Recio, Sommer, & Wilhelm, 2014; Niedenthal, 2007; Rychlowska et al., 2014; Schilbach, Eickhoff, Mojzisch, & Vogeley, 2008]. Our results suggest that the effect of JA on SFM is dependent on autistic traits. Thus, we speculate that in ASC not only the different domains of empathy by themselves might be impaired [Edwards, 2014; McIntosh et al., 2006; Nation & Penny, 2008; Oberman et al., 2009], but also the interaction between them might be affected.

Due to technical constraints, we could only record from one muscle (Zygomaticus Major) in this study. Our decision to focus on the Zygomaticus Major only was based on the large body of literature on the spontaneous mimicry of happy expressions [Niedenthal et al., 2010]. Future studies should test if this result extends to the spontaneous mimicry of negative emotions, such as anger. Additionally, it would be informative to test the impact of gaze following per se on SFM, and if autistic traits modulate this impact. The current experiment does not allow for such an inference, as all conditions required gaze following. A limitation of the study is the lack of a significant effect of expression. Although Zygomaticus Major activity was higher for the happy compared to the disgust condition, this difference did not reach significance.

To control for potential gender effects, we correlated AQ and JA‐NoJA difference scores separately for each gender and found that the negative relationship was only significant in males, indicating that the modulatory effect of JA might be more strongly related to AQ in male participants. Potential explanations for this could be greater motives for affiliation [Brody & Hall, 2008] and a generally higher social sensitivity and fewer sociocommunicative difficulties in females in comparison to males [Connellan, Baron‐Cohen, Wheelwright, Batki, & Ahluwalia, 2000; McClure, 2000]. These abilities have been reported to be more preserved in females with ASC [Lai et al., 2011]. These factors might prevent an atypical response to JA in females high in autistic traits.

The paradigm employed in this study has been used successfully to study JA before [Schilbach et al., 2010; Wilms et al., 2010]. Because of its interactive and gaze‐based nature it resembles real‐world social interactions and should, therefore, be superior to paradigms presenting emotional facial expressions in form of static pictures or videos. Nevertheless, one cannot rule out artefacts of a virtual interaction that would not generalize to real life interactions. In summary, this study provides a direct test of how JA influences SFM, and in particular, the critical role of autistic traits in shaping this relationship. These findings suggest that atypical interactions between JA and SFM, in addition to testing these processes individually, might be crucial to understanding the nature of social deficits in autism.
